# Genome-Wide Analysis of Tea FK506-Binding Proteins (FKBPs) Reveals That *CsFKBP53* Enhances Cold-Stress Tolerance in Transgenic *Arabidopsis thaliana*

**DOI:** 10.3390/ijms26083575

**Published:** 2025-04-10

**Authors:** Ming-Hui Xu, Jie Tang, Cai-Ning Liu, Wan-Qiao Zhang, Qian Li, Fan Yang, Dan-Dan Liu

**Affiliations:** 1 School of Agriculture, Yunnan University, Kunming 650091, China; xmh199802@163.com (M.-H.X.); olyyo3@163.com (J.T.); liucaining@mail.ynu.edu.cn (C.-N.L.); zhang765710192@163.com (W.-Q.Z.); liqian1717@163.com (Q.L.); 2 Yunnan International Joint R&D Center for Intergrated Utilization of Ornamental Grass, College of Landscape and Horticulture, Southwest Forestry University, Kunming 650224, China

**Keywords:** *Camellia sinensis*, *FKBP* gene family, *CsFKBP53*, function analysis, low temperature

## Abstract

FK506-binding proteins (FKBPs) belong to the peptidyl-prolyl cis/trans isomerase (PPIase) superfamily and are involved in a wide range of biological processes including protein folding, hormone signaling, plant growth, and stress responses. However, the FKBPs and their biological functions have not been identified in tea plants. In this study, 21 *FKBP* genes were identified using the conserved FK506-binding domain (PF00254) in the tea-plant genome. Their phylogeny, classification, structure, motifs, interactors, and expression patterns were analyzed. Comprehensive qRT-PCR analysis revealed distinct expression patterns of *CsFKBPs* in different tissues and in response to low temperature. Through a comprehensive genome-wide analysis, we characterized the low-temperature expression dynamics of the *CsFKBP53* gene family and demonstrated that its overexpression significantly enhances cold tolerance in *Arabidopsis*. Notably, the transcript levels of *CsFKBP53* exhibited pronounced variability across distinct tea (*Camellia sinensis*) cultivars under cold-stress conditions. These findings not only underscore the functional conservation of FKBP-type immunophilins across plant lineages but also highlight the biotechnological potential of CsFKBP53 as a genetic modulator of low-temperature resilience in crops. By integrating comparative genomics and functional validation, our study establishes a foundation for leveraging conserved stress-response mechanisms to engineer climate-resilient plants.

## 1. Introduction

Immunosuppressants are defined as receptors for immunosuppressive drugs such as cyclosporine A (CsA), tacrolimus (FK506), and rapamycin (sirolimus). FKBPs are distinguished by their peptidyl-prolyl cis-trans isomerase (PPIase) enzymatic activity, which catalyzes the conformational rotation of proline-containing peptide bonds between cis and trans states, a critical process in protein folding [1,2,3]. Structurally, all FKBP family members share a conserved FK506-binding domain (FKBd/FKBP-C), typically spanning ~110 amino acids. This domain not only houses the catalytic site for PPIase activity but also serves as the ligand-binding pocket for proline residues and synthetic analogs, underscoring its dual role in enzymatic catalysis and pharmacological interactions [4].

Advances in genome assembly and bioinformatics have enabled the identification and characterization of *FKBP* family genes across diverse plant species, yet their roles in tea plants (*C. sinensis*) remain underexplored. Some *FKBP* genes have been found to play an important role in abiotic stresses in different plants. In *Arabidopsis*, the FKBP62 (ROF1) and FKBP65 (AtROF2) function as reciprocal regulators of heat-stress tolerance. AtROF1 interacts with HSP90 via its tetratricopeptide repeat (TPR) domain, facilitating the formation of a ternary complex with the heat-shock transcription factor HsfA2 that drives the expression of small heat-shock proteins (sHSPs) to mitigate heat damage [5]. Conversely, AtROF2 antagonizes this response by binding to AtROF1’s FKBd, disrupting the complex and repressing sHSP synthesis [5]. Consistent with their opposing roles, *AtROF1* knockdown plants exhibit severe heat sensitivity, phenocopying *HsfA2* mutants, while *rof2* mutants or *AtROF1*-overexpressing lines show enhanced thermotolerance [6,7]. *AtFKBP20-1a* was highly expressed in all tissues and caused a rapid increase in expression under both heat and drought stress, but *AtFKBP20-1b* expression only began to increase after 24 h of stress treatment. Both were localized in the nucleus, while AtFKBP20-1b was also present in the cytoplasm [8]. AtFKBP15-1 and AtFKBP15-2, which are localized to the endoplasmic reticulum in *Arabidopsis*, are 70% homologous and both are induced by high temperature [9]. In *Zea maize*, FKBPs (ZmFKBPs) play a critical role in cold-stress signaling pathways. Notably, transcript levels of *ZmFKBP12*, *ZmFKBP13*, *ZmFKBP15-3*, *ZmFKBP16-1*, and *ZmFKBP57* exhibited rapid and sustained repression under chilling conditions (4 °C) [10]. These findings suggest that specific FKBP family members may function as positive regulators in the plant’s adaptive response to low-temperature stimuli.

The FKBPs in plants and diverse eukaryotic organisms exhibit complex structural architectures beyond their canonical FKBd (FK506-binding domain). These multi-domain scaffolds integrate several functional modules, such as the tetratricopeptide repeat (TPR) domain—which forms inverse–parallel α-helical arrays essential for mediating protein–protein interactions within multi-component complexes and specifically associates with heat-shock protein 90 (HSP90) alongside calmodulin-binding domains (CaMBDs) that modulate calcium-dependent signaling pathways, Novel Plant-specific (NPL) domains with undefined biological roles, and a Trigger factor (TIG) domain implicated in protein-folding chaperone functions. CaMBDs are generally located at the C-terminus of plant bulk FKBP and can bind to the calmodulin CaM (calmodulin), the C-terminal CaMBD can be regulated by Ca^2+^ ion signaling to activate its PPIase and molecular chaperone activity, suggesting that multi-structured plant FKBPs may have similar calcium regulation mechanisms [11]. Recent investigations have unveiled a functional NPL structural domain within the *Drosophila FKBP* gene family that specifically interacts with core histones, thereby facilitating nucleosome assembly and chromatin organization [12]. The TIG proteins represent a class of multi-domain triggers closely associated with FKBPs, characterized by a bipartite structural organization comprising an FKBD and a C-terminal chaperone domain. The central FKBD is flanked by N-terminal ribosomal interactions, exhibiting peptidyl prolyl cis/trans isomerase activity analogous to FKBP-C [13,14,15], which contributes to protein translocation processes. Although it has been demonstrated that TIG can act as a molecular chaperone in prokaryotes interacting with nascent polypeptides in an open conformation, its role in plants is not yet clearly recognized. It is suggested that TIG might be involved in plant chloroplast translation and protein folding [16,17]. It is indisputable that the multi-structural domain FKBP will also undertake more complicated and important functions due to its complex structure.

Tea is an important beverage plant with economic, health, and cultural value [18]. Tea trees in China are preferentially cultivated in mountainous regions, where high-elevation environments are often perceived to enhance tea quality. Such cultivation practices can inadvertently expose plants to suboptimal thermal conditions and insufficient light exposure during critical growth phases, thereby increasing their vulnerability to cold snaps and frost events [19,20]. Meanwhile, the drop in temperature in spring when the buds are sprouting can also seriously affect the yield of tea plants. These low-temperature stress phenomena, caused by a combination of geographic selection and climatic fluctuations, have not only restricted the range of suitable areas for the tea tree, but also reduced the yield and quality of the tea [21], Identifying relevant genes to cope with low temperatures and breeding new tea-plant varieties with excellent cold-resistant traits to improve tea production have become key scientific problems to be solved in the field of the genetic improvement of tea plants. With the publication of genomic data for important tea-tree varieties such as “Shuchazao” (*C. Sinensis* var. *Assamica* cv. *Shuchazao*, Shuchazao) and “Yunkang10” (*C. Sinensis* var. *Assamica* cv. *Yunkang10*, Yunkang10) [22,23,24], the systematic identification and mining of resistance gene families became possible, and provided a framework for molecular studies to resolve the synergistic regulatory network of biotic and abiotic stresses in tea plants. The aim of this study is to analyze the molecular regulatory network of tea plants in response to low-temperature stress, and to reveal the biological roles of key functional genes in the cold-resistance mechanism of plants. In view of the lack of genetic resources in the creation of cold-resistant tea germplasm, we explore low-temperature responsive elements with potential for breeding applications. In this study, using the Yunnan large-leafed tea cultivar “Yunkang10” with excellent cold resistance as a material, we screened *CsFKBP53*, a key gene for cold-stress resistance, and its expression pattern and the phenotypic traits of transgenic *Arabidopsis* were analyzed to investigate the cold-resistance function. To provide theoretical support for the creation of new highly cold-resistant and high-quality tea germplasm, as well as molecular-marker-assisted selection breeding.

## 2. Results

### 2.1. Identification and Physical Properties of CsFKBPs

To identify encoding FK506-binding proteins in the Yunkang10 genome, we used an FK506-binding sequence to identify, and the results were retrieved from HMMER, PFAM, SMART, and CD-Search. We identified 21 CsFKBPs in the database. We named the CsFKBPs according to their molecular weight and homology with the *Arabidopsis* and rice *FKBP* genes, based on the nomenclature rules of previous studies [25,26,27]. The gene name, gene ID, CDS length, number of free amino acids, molecular weight, theoretical PI, subcellular localization, and the number of FKBP-C domains are shown in Table 1. The *FKBP* genes in tea consist of 100–619 amino acids, with CDS lengths between 306 bp and 2429 bp, molecular weights between 10.81 kDa–72.43 kDa, and theoretical PI between 4.5 and 10.44. Among them, 57% of the CsFKBPs are basic. Subcellular localization predictions showed that they were located in the nucleus, chloroplast, cytoplasm, vesicles, peroxisomes, and extracellular matrix, with 38% of the genes located in the nucleus and 33% in the chloroplast, indicating that CsFKBPs may function mostly in the nucleus and chloroplast.

**Figure 1:**
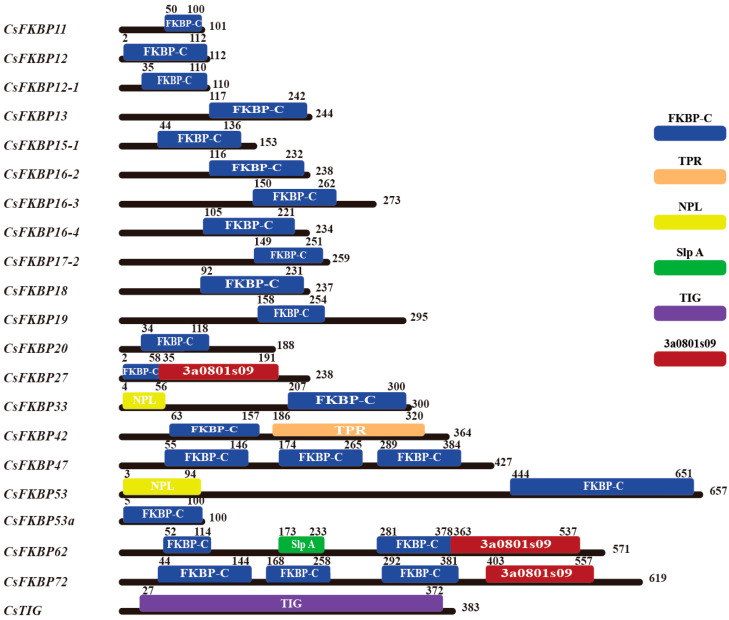
A structural domain analysis of CsFKBPs. The functional domain distribution of 21 FKBPs in tea. We used the CD-Search program to identify the six conserved domains shown in the colored boxes in the CsFKBP protein sequence. Blue box: FKBP-C domain; orange box: TPR domain; yellow box: NPL domain; green box: Slp A domain; purple box: Trigger_C domain; and red box: 3a0801s09. The functional domains are arranged in an order that corresponds to their position in the sequence of the individual protein sequence. The numbers in the figure reflect their position in the protein sequence.

The 21 CsFKBPs contain 6 different structural domains, of which 13 genes contain only one FKBP-C domain, and CsFKBP62 with two, and CsFKBP47/72 with three (Table 1, Figure 1). In contrast to other species, the TPR domain is only found in CsFKBP42 and the position is similar to that of FKBPs found in other species. The Tigger_C domain is only found in CsTIG, but it does not contain the FKBP_C domain. The NPL domain is found at the C-terminus of CsFKBP33 and CsFKBP53. CsFKBP27, CsFKBP62, and CsFKBP72 contain a 3a0801s09 domain at the N-terminus. Unfortunately, the function of this domain is not well explored at present.

We obtained information on the chromosomal distribution of the 21 *CsFKBP* genes, as shown in Supplementary Table S1. Genetic maps of 21 *CsFKBP* genes were created using the online tool MG2Cv2.1. They were distributed on 10 chromosomes: Chr01, Chr03, Chr04, Chr05, Chr06, Chr08, Chr10, Chr11, and Contig235 (Supplementary Table S3, Figure S1).

### 2.2. Evolutions of FKBPs in Different Species

**Figure 2:**
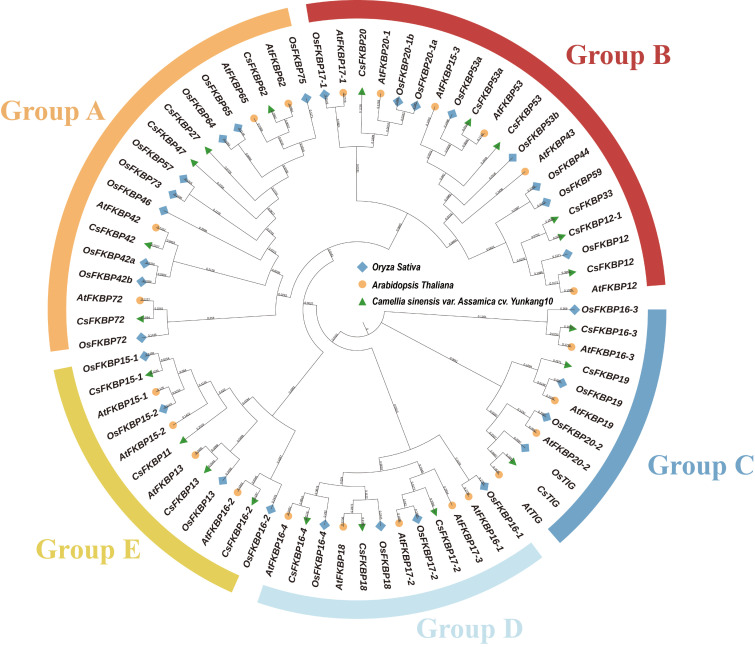
A phylogenetic and evolutionary analysis of FKBPs. Phylogenetic trees were generated using the neighbor-joining method based on the full-length amino-acid sequences of FKBP in different species, including Yunkang10, *Arabidopsis*, and rice. The different colors and symbols represent different species. Blue box: rice; orange circle: *Arabidopsis*; green triangle: Yunkang10.

It is possible to predict the function of some of the CsFKBPs by examining the classification results of the evolutionary tree and the role of each subfamily member in other plants. For example, CsFKBPs in group B may play an important role in the resistance of tea plants to low-temperature stress [10]. To explore the evolutionary relationships among tea plants and the *FKBP* genes of *Arabidopsis* and rice, we built a phylogenetic tree using the full-length amino-acid sequences of the tea plants, *Arabidopsis*, and rice *FKBP* genes, of which there are 21, 23, and 29 *FKBPs* in tea plants, *Arabidopsis*, and rice, respectively (Figure 2). The results demonstrated that the 73 *FKBP* genes were classified into five groups: A, B, C, D, and E, based on structural domains and evolutionary relationships. Six (28.5%) of the FKBP genes in Yunkang10 were classified in group B, which contained the group containing the most *FKBP* genes in tea. Additionally, groups A, C, D, and E contained 5, 3, 4, and 6 *CsFKBPs*, respectively. Groups A and B were located the same branch, which may indicate that the genes in these two groups are more closely related genetically.

### 2.3. Gene-Structure and Conserved Motif Analysis of CsFKBPs

**Figure 3:**
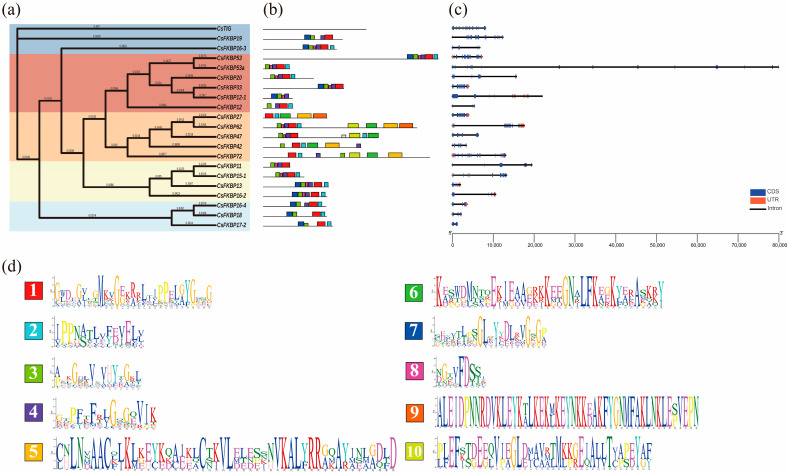
The evolutionary tree, conserved motif distribution, and gene structure of *CsFKBP* genes. (**a**) The evolutionary tree was built based on the 21 CsFKBPs protein sequences. (**b**) Conserved motifs of the members of the CsFKBPs. (**c**) The structure of CsFKBPs. Blue for CDS, orange for UTR, and straight lines for introns. (**d**) Ten conserved motif structures of the CsFKBP protein in tea. The ordinate indicates amino-acid conservation, and the height of amino-acid letters indicates the frequency of occurrence. The abscissa represents the position of the amino acid in the sequence.

An integrated analysis of conserved structural motifs and hierarchical clustering patterns revealed distinct phylogenetic groupings among homologous family members (Figure 3a). This analysis demonstrated that genes sharing similar functional domains exhibited coherent clade formation, indicating evolutionary conservation within this gene family. We identified 10 motifs in 21 *CsFKBPs* through an analysis using the MEME Suite 5.5.7 online software (Figure 3b,d). The results showed that except for CsTIG, other CsFKBPs contain multiple motifs. Motif 1 and motif 3 are present in every CsFKBP, presumably as the most conservative and basic motifs of the FKBP family. It is noteworthy that CsFKBPs in groups B, C, D, and E exhibited a high degree of similarity in their motif domains, except CsTIG in group C. Gene-structure information shows that members of the CsFKBP family all contain introns and exons, including at least 2 and up to 19 introns (Figure 3c). However, eight genes do not have an untranslated region (UTR). All genes contain multiple coding sequences (CDSs) except for *CsFKBP17-2*, which contains two CDSs.

### 2.4. Expression Patterns of CsFKBPs Under Different Cold Stresses

**Figure 4:**
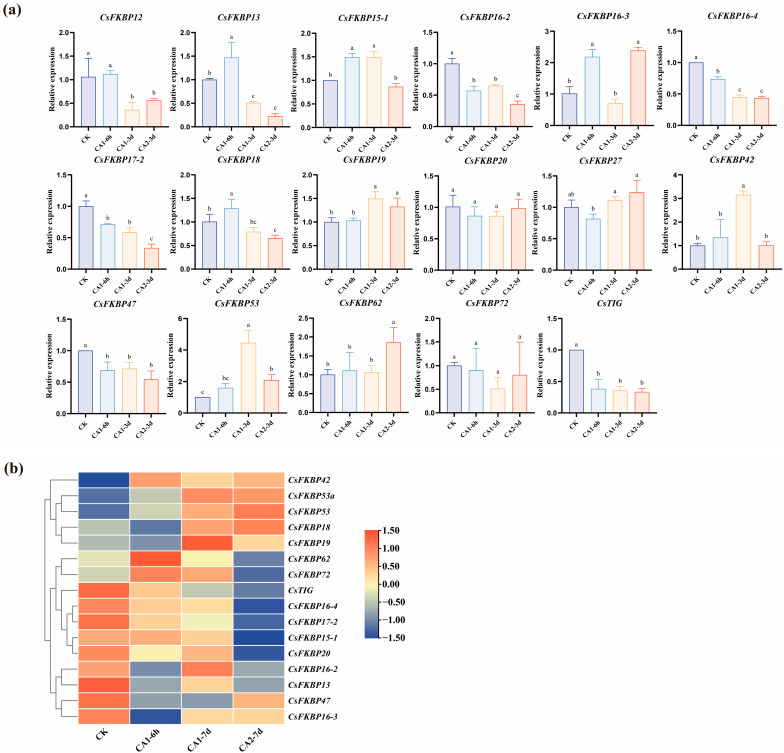
The expression of *CsFKBPs* under different cold stresses. (**a**) The expression pattern of *CsFKBPs*. CK was the control, CA1-6h was 6 h of 10 °C stress, CA1-3d was 3 days of 10 °C treatment, and CA2-3d was 3 days of 4 °C treatment (*p* < 0.05). The different lowercase letter markers represent significant differences, and the significance level is taken as 0.05. (**b**) A heat map of the expression of *CsFKBPs* in Shuchazao.

We analyzed the expression of 21 *CsFKBPs* in Yunkang10 old leaf tissues by qRT-PCR, among which four members had no expression detected: *CsFKBP11*, *CsFKBP12-1*, *CsFKBP33*, and *CsFKBP53a*, respectively (Figure 4a). The results showed that the expression trends of different members were variable. Genes that responded significantly to low-temperature stress included *CsFKBP16-3*, *CsFKBP42*, and *CsFKBP53*, which showed an overall increasing trend in expression under more severe low-temperature conditions, indicating that they may exercise important functions when subjected to low-temperature stress. Among them, *CsFKBP53* showed an increase in expression after both low-temperature stresses and reached 2-fold the amount of CK after 6 h and 3 days at 4 °C and even more than 4-fold after 3 days of treatment at 10 °C. In addition, we compared the expression patterns of homologous genes in Shuchazao, formed a heat map, and found that their expression profiles were similar (Figure 4b). The results indicate that the biological function of *CsFKBP53* in response to low-temperature stress in different tea plants is highly conserved.

### 2.5. Sequence and Phylogenetic Analyses and Subcellular Localization of CsFKBP53

**Figure 5:**
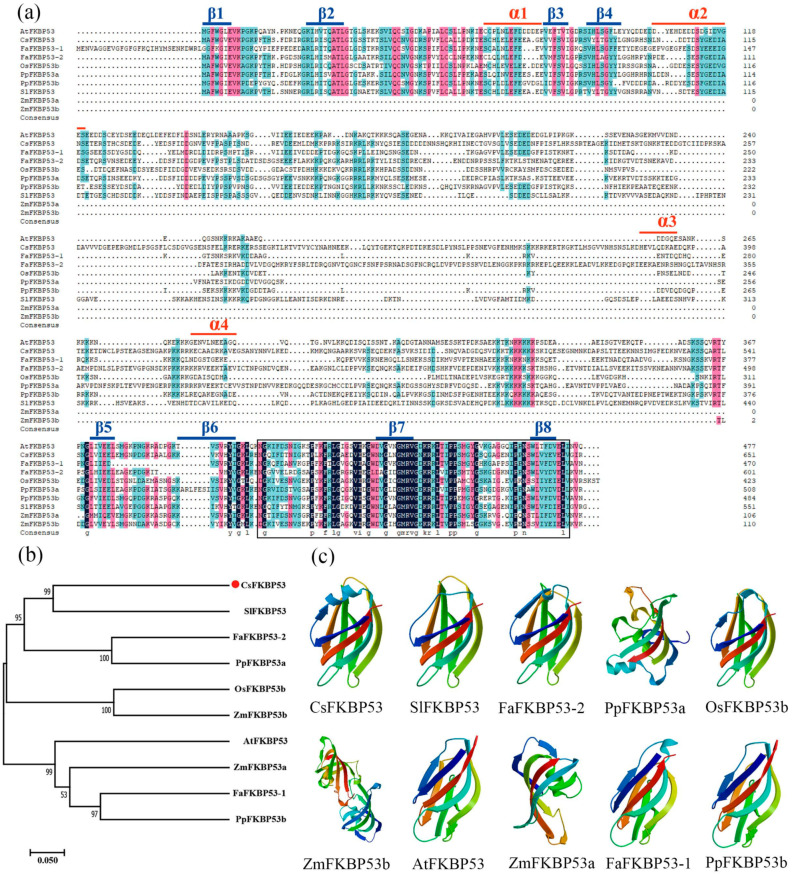
Multiple sequence alignment, cluster analysis, and protein-structure analysis of the conserved structural domains of FKBP53 proteins. (**a**) Multiple sequence alignment of CsFKBP53 with homologous proteins of other species. α-helices and β-folds are marked above the sequence with red and blue straight lines, respectively. The FKBP-C structural domain is displayed with in the black box, with the same amino acids represented on a dark blue background. (Similarity: dark blue = 100%; pink > 75%; cyan > 50%) (**b**) A phylogenetic analysis of CsFKBP53 and other species with homologous proteins. (**c**) The three-dimensional structure of FKBP53 with homologous proteins of other species. Different colours represent different folding styles.

To investigate the evolutionary relationship between FKBP53 homologs in different species, we compared the reported FKBP53 sequences in *Arabidopsis*, rice, strawberry, peach, tomato, maize, and tea (Figure 5a). The results show that the FKBP53s all contain an FKBP-C structural domain and multiple amino-acid sites are conserved. In addition, we also analyzed the affinities among CsFKBP53 and FKBP53 of other species, and found that CsFKBP53 was the closest to SlFKBP53, with a homology of 43.21% (Figure 5b). The results of three-dimensional structure analysis showed that the three-dimensional structures of the seven proteins, CsFKBP53, Sl-FKBP53, FaFKBP53-2, PpFKBP53a, OsFKBP53b, ZmFKBP53b, AtFKBP53, ZmFKBP53a, FaFKBP53-1, and PpFKBP53b, were all mainly composed of β-folding, and all of them showed a highly similar state (Figure 5c). These results may indicate that FKBP53 proteins in different species may have similar functions.

### 2.6. The Expression Pattern Analysis and Subcellular Localization of CsFKBP53

**Figure 6:**
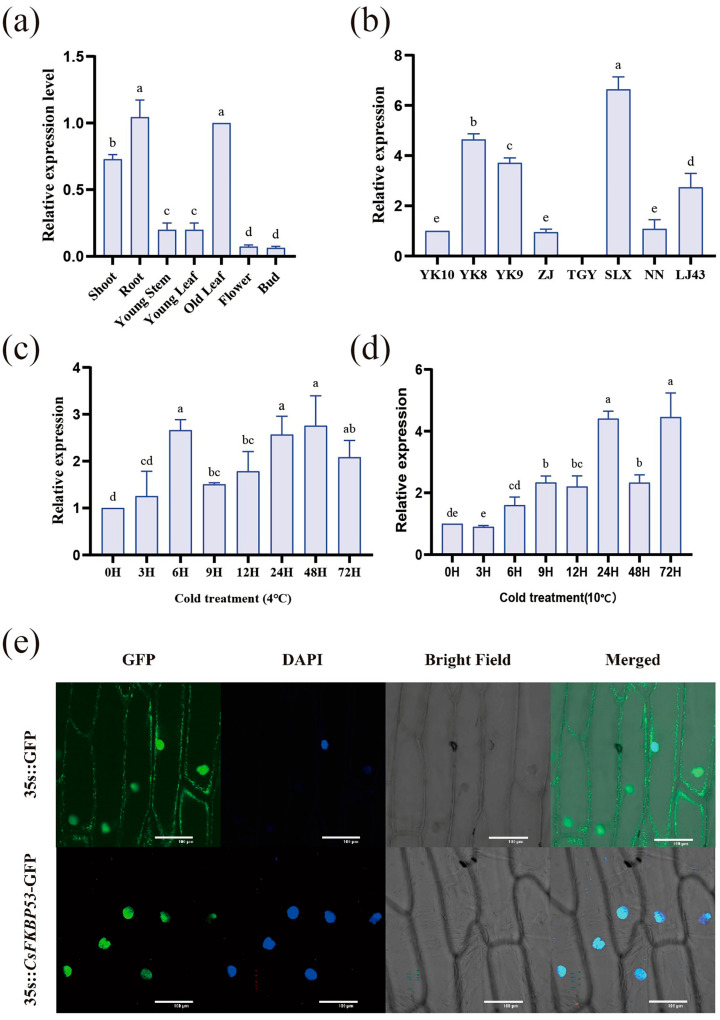
The relative expression and subcellular localization of *CsFKBP53*. (**a**) The relative expression levels of *CsFKBP53* in different tissues. (**b**) The relative expression levels of *CsFKBP53* in different tea species. (**c**,**d**) The relative expression levels of *CsFKBP53* after 4 °C and 10 °C cold stress. (**e**) The localization of *CsFKBP53*-GFP in the lower epidermal cells of round onions was observed by laser confocal microscopy using 35s-GFP as a control. GFP: green fluorescence channel; Bright Field: bright field; Merged: GFP and bright field merged channel. Scale bar = 100 μm. The different lowercase letter markers represent significant differences, and the significance level is taken as 0.05.

We analyzed the expression of *CsFKBP53* in different tissues of Yunkang10 (YK10) and in old leaf tissues of different tea trees, including Yunkang8 (YK8), Yunkang9 (YK9), Zijuan (ZJ), Tieguanyin (TGY), Shilixiang (SLX), Nannuodayecha (NN), and Longjing43 (LJ43). The results showed that *CsFKBP53* was highly expressed in the tissues of roots and old leaves, and it has a high degree of tissue-expression specificity (Figure 6a,b). The expression was about 3 times higher than that in young stem and young leaf tissues, and about 5 times higher than that in flowers and buds; the highest expression was found in the highly cold-resistant varieties Shilixiang, Yunkang8, and Yunkang9, whereas no expression signals were detected in Tieguanyin. We also found that *CsFKBP53* expression was up-regulated after 10 °C and 4 °C treatments (Figure 6c,d). During the 10 °C treatment, the difference between the 3 h treatment and the 0 h control was not obvious. However, the expression increased significantly after 24 h. The expression pattern was also up-regulated in the 4 °C treatment relative to the control. During the 4 °C treatment, the expression peaked at 24 h, 48 h and 72 h, which proved the low-temperature correlation of *CsFKBP53*.

*CsFKBP53* was transformed into the onion’s lower epidermal tissues by constructing a *CsFKBP53-GFP* fusion-protein transient expression vector, and the fusion protein was observed to be localized in the nucleus of the cells using laser confocal microscopy (Figure 6e), while the control exhibited a green fluorescent signal in the cell, including the nucleus and cells.

### 2.7. Transgenic CsFKBP53 Improves Low-Temperature Resistance of Arabidopsis

**Figure 7:**
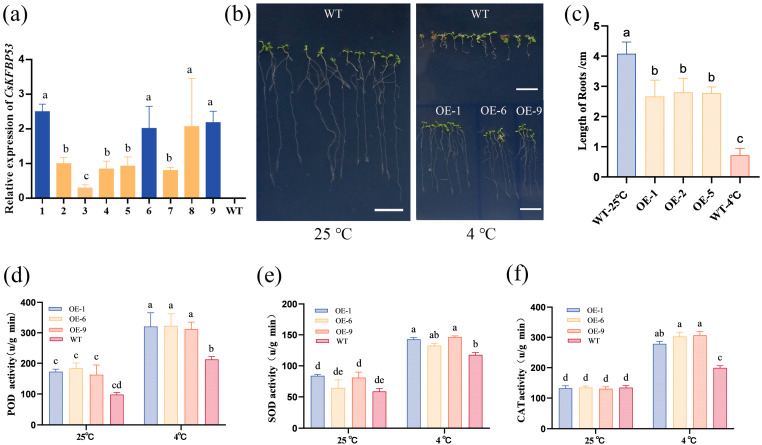
Cold sensitivity of CsFKBP53-OE plants. (**a**) Expression analysis of CsFKBP53 transgenic plants. The expression of CsFKBP53 transgenic plants was analyzed by qRT-PCR. Leaf tissues from nine overexpression lines and wild-type control WT were collected for qRT-PCR analysis. (**b**) Phenotypic comparison of *Arabidopsis* plants. WT-4 °C is the 4 °C-treated 48 h wild-type control, OE-4 °C is the 4 °C-treated 48 h CsFKBP53 overexpression plants, and WT-25 °C is the 25 °C wild-type control. Scale bar = 1 cm. (**c**) *Arabidopsis* plant root-length statistics, three replicates of each plant were taken for measurement, and the vertical line in the figure indicates the standard error. A significance analysis was performed using SPSS 22 for one-way ANOVA, and different lowercase letters indicate significant differences. (*p* < 0.05) (**d**–**f**). The results of POD, SOD, and CAT activity assays in *Arabidopsis* plants.

To further validate the cold-resistant function of *CsFKBP53*, it was transfected into wild-type *Arabidopsis* by heterologous expression, and a total of nine overexpression lines were obtained and their expression was examined (Figure 7a). The results showed that no fluorescent signals were detected in the wild-type control (WT), whereas the expression of *CsFKBP53* was highly expressed in the overexpression lines OE-1, OE-6, and OE-9. In addition, we cultured T2 generation seedlings and WT plants and placed them in a 4 °C incubator after one week of germination for 48 h of low-temperature stress treatment, while 10 WT plants cultured at 25 °C under ambient conditions were taken as the control, and the phenotypic changes in the growth process were observed and recorded (Figure 7b). The results showed that the phenotypes of the plants changed significantly after low-temperature treatment. Under low-temperature (4 °C) conditions, wild-type *Arabidopsis* (WT) exhibited significant morphological alterations compared to optimal growth temperature (25 °C), including leaf-margin curling, purple pigmentation in rosette leaves, and severely impaired root development. In contrast, transgenic plants overexpressing the target gene maintained normal growth patterns post cold acclimation, demonstrating enhanced cold tolerance. A quantitative analysis of root elongation revealed a distinct hierarchy: 25 °C WT > 4 °C OE > 4 °C WT (Figure 7c). These results indicate that low-temperature treatment drastically inhibited root growth in wild-type plants, while transgenic lines exhibited robust root development under identical stress conditions, suggesting effective mitigation of low temperature-induced growth repression through genetic modification.

The T3 generation transgenic plants were subjected to a low-temperature treatment of 4 °C for 24 h to detect changes in the contents of the peroxidase (POD), superoxide dismutase (SOD) and catalase (CAT) indices. The results showed that relative to 25 °C WT, POD activity (173.09–318.55), SOD activity (76.38–140.52), and CAT activity (132.44–296.04) were significantly increased in the three transgenic lines after 4 °C low-temperature treatment, and showed a more obvious increasing trend than WT (Figure 7d–f). The results suggest that CsFKBP53 may improve cold tolerance in *Arabidopsis* by increasing the activity of antioxidant enzymes, eliminating the accumulation of ROS and reducing cellular damage.

## 3. Discussion

In recent years, some researchers have identified the *FKBP* (FK506-binding protein) genes in *Arabidopsis* [28,29,30], rice [31,32,33], apple [25], strawberry [34], tomato [13], grape [35], peach [36], and maize [10], and found that they play important functions in plant growth and development as well as in abiotic stress response. They were found to play important functions in plant growth and development as well as in abiotic stress response. However, as an economically important plant, the *CsFKBP* family of genes in tea trees has not yet been reported. In the present study, a bioinformatic analysis revealed that tea and peach had the same number of FKBP family members, with 21 family members (Table 1). Whereas *Arabidopsis*, rice, strawberry, and grape had 23, tomato and maize had 24, and apple had 28. The difference in the number of FKBP family members may indicate that the *FKBP* family genes have undergone genetic divergence during the evolution of different species. The differences in the number of FKBP family members may indicate that gene duplication or loss events have occurred during the evolution of different species. There is a wide range of variation in the size of plant FKBP family members; for example, *AtFKBP12* is a single domain (SD) FKBP consisting of only one FKBd, whereas wheat *TaFKBP77* is an FKBP with multiple structural domains (multi-domain, MD) [37]. In this study, the length of the 21 *CsFKBPs’* CDSs ranged from 306 bp (*CsFKBP11*) to 2429 bp (*CsFKBP53a*), encoding amino-acid sequences ranging from 100 aa to 619 aa, with a large variability among the different CsFKBPs.

Moreover, in addition to the FKBP-C domain, other structural domains have been identified in the FKBP family: the tetratricopeptide repeat (TPR), the calmodulin-binding domains (CaMBDs), Tigger-C, and NPL. These structural domains have complex functions and may be involved in protein complex assembly, stimulating other structural domains, promoting chloroplast translation, and participating in protein folding. The results of this study show that tea-tree CsFKBP protein is similar to other species in that they contains FKBP-C, TPR, and TIG structural domains (Figure 1). Seventeen of these proteins contained one FKBP-C domain and three contained multiple FKBP-C domains. However, the Slp A and 3a0801s09 domains in CsFKBPs have not been reported in other species, and their specific functions need to be further investigated. Interestingly, motif 3 is present in all FKBP members except CsTIG (Figure 3b). This indicates that motif 3 corresponds to the FKBP-C structural domain, which is the most conserved segment of the FKBP-C structural domain of CsFKBPs. To investigate the clustering of FKBPs in tea, *Arabidopsis*, and rice, a phylogenetic tree was constructed based on the amino-acid sequences of FKBPs from each species. A total of 73 proteins were divided into five groups, with CsFKBPs having the most distributed members in Group B (Figure 2 and Figure 3a). The 21 CsFKBPs were also clustered and found to be classified into seven groups. The clustered CsFKBPs had similar gene structures and conserved structural domains, and this result was similar to the clustering results of *Arabidopsis* FKBPs [30].

The temperature of 10 °C is the limit at which tea plants are subjected to low-temperature stress, while tea plants are already under low-temperature stress when the temperature is below 4 °C [38,39]. Transcriptome analysis showed that the *CsFKBP* family of genes in the tea tree showed a more pronounced expression trend under low temperature stress conditions. We also examined the expression of *CsFKBPs* at different periods of 4 °C treatment and 10 °C treatment by qRT-PCR (Figure 4a). The results showed that these genes showed different trends with increasing times of low-temperature treatment, with the most obvious trend of increasing *CsFKBP53* expression. A further analysis of the spatial and temporal expression patterns of *CsFKBP53* under low-temperature stress showed that *CsFKBP53* expression began to show an increasing trend after 3 H treatment at 10 °C, and reached the highest expression at 24 H and 72 H. The response of *CsFKBP53* to low temperature was more rapid at 4 °C treatment, and the expression reached a peak at 6 H treatment. The expression of *CsFKBP53* reached the peak at 6 H and then began to decrease, but the expression increased to about 2.5 times that of the control group at 48 H (Figure 4b). In conclusion, *CsFKBP53* showed a positive trend in response to low temperature stress, and its expression increased more positively at lower growth temperatures.

Zhao [40] found that the low-temperature stress-response gene *CsWRKY37* was most highly expressed in the roots of tea plants. In contrast, *CsFKBP53* also showed higher expression in more lignified roots in the tissue-expression pattern analysis of Yunkang 10. The results of the tissue-expression pattern analysis may prove that the low-temperature stress-related genes are more biased to exercise their functions in tea-tree roots that are less damaged under low-temperature conditions (Figure 6a). The results of Wang [41] et al. found that tea-tree varieties with a common leaf color were more cold-resistant than purple leaf varieties, with Longjing 43 showing stronger cold resistance while Zijuan tea was less cold resistant. The Yunkang species varieties are a national breed selected by the Yunnan Agricultural Institute through asexual lines and are generally highly resistant to cold and can be grown at an absolute temperature of −5 °C. The low-temperature correlation was verified by the higher expression of *CsFKBP53* in the old leaf tissue of the highly cold-resistant tea-tree varieties (Figure 6b). The expression showed a positive correlation with the cold resistance of tea-tree varieties.

Plants must regulate various physiological and biochemical processes to alleviate low-temperature stress [42]. Low-temperature stress reduces the growth rate of plants, which is reflected in the phenotypic effects of low-temperature stress on leaf growth and root-growth vigor [43]. In terms of the enzymatic activity of antioxidant enzymes, low-temperature stress resulted in excessive accumulation of ROS, which in turn promoted the expression of a series of antioxidant enzyme genes and induced an increase in the content of catalase (CAT), peroxidase (POD), and superoxide dismutase (SOD) in the plant to eliminate ROS accumulation to avoid cell damage [44]. In this study, we successfully transferred *CsFKBP53* into wild-type *Arabidopsis* and obtained three highly expressed transgenic lines (Figure 7a). The growth of the three transgenic lines under low-temperature stress was significantly better than that of the wild-type control, as evidenced by longer root length and reduced leaf purplishness (Figure 7b,c). In addition, the results of POD, SOD, and CAT assays showed that the activities of all three physiological indicators were significantly increased after the transgenic *CsFKBP53 Arabidopsis* plants were treated with low temperature at 4 °C for 24 h (Figure 7d–f). This indicated that the transgenic *CsFKBP53* enhanced the activity of POD, SOD, and CAT in *Arabidopsis* and improved the permeability of cell membranes, which ultimately led to the enhanced cold resistance of the transgenic plants.

## 4. Materials and Methods

### 4.1. Plant Materials and Stress Treatments

To evaluate the functions of FKBPs, two-year-old “Yunkang10” was used as the plant material, from a horticulture greenhouse at Yunnan University (Yunnan, China). Different tea-plant components including shoots, roots, stems, young leaves, old leaves, flowers, and buds were collected, immediately frozen in liquid nitrogen, and stored at −80 °C. Collected “Yunkang8” (*C. Sinensis* var. *Assamica* cv. *Yunkang8*, Yunkang8), “Yunkang9” (*C. Sinensis* var. *Assamica* cv. *Yunkang9*, Yunkang9), “Longjin43” (*C. Sinensis* var. *Sinensis* cv. *Longjin43*, Longjin43), “Tieguanyin”, “Zijuan” (*C. Sinensis* var. *Assamica* cv. *Zijuan*, Zijuan), “Nannuo”, and “Shilixiang” old leaf tissues were stored at −80 °C. *Arabidopsis* seeds were grown in a greenhouse in a controlled environment for 16 h per day (25 ± 3 °C) and 8 h at night (20 ± 3 °C) at 70% relative humidity. Take a batch of YK10 tea plant, part of which was treated at low temperature (4 °C/10 °C) in a low-temperature incubator, and the rest were used as controls. Collect it at different times (0, 3, 6, 9, 12, 24, 48, and 72 h), immediately freeze in liquid nitrogen, and then store it at −80 °C for RNA extraction. *N. benthamiana*, used in this study, was grown in growth chamber pots at 20 °C at night and 24 °C during the day, with a photoperiod set to 16 h of light and 8 h of darkness.

### 4.2. Identification and Physical Properties of FKBP Genes in Tea

The genome sequencing and assembly of the tea plant Yunkang 10 were completed, and the protein and CDSs were published in TPIA (https://tpdb.shengxin.ren/index.html (accessed on 10 August 2022)) [45,46]. Hidden Markov model profiles in the FK506-binding domain were downloaded from the Pfam database. Sequence information for the *AtFKBP* and *OsFKBP* genes was obtained from the TAIR (https://www.arabidopsis.org/, accessed on 7 April 2025), and RGAP websites, respectively. The hidden Markov model profile in the FK506-binding domain (PF00254) was downloaded from the Pfam (https://pfam.xfam.org/, accessed on 7 April 2025), The HMMER V3.3.2 (http://hmmer.org/, accessed on 7 April 2025) was used to search through the amino-acid sequences of the Yunkang10 genome (e value < 10^−5^) and the sequences were used as candidate sequences for the FKBP gene of tea [47]. The above sequences were used as candidates for the FKBP gene of the tea tree, and the structural domains were verified using the SMART [48] (http://smart.embl.de/, accessed on 7 April 2025) and the CD-Search database in NCBI (https://www.ncbi.nlm.nih.gov/Structure/cdd/wrpsb.cgi, accessed on 7 April 2025), respectively. The physicochemical properties such as isoelectric point, molecular weight, and theoretical PI values of all members of the *CsFKBP* gene family were predicted using ExPASY (https://web.expasy.org/protparam/, accessed on 7 April 2025). Chromosome localization analysis was carried out using the online site MG2Cv2.1 (http://mg2c.iask.in/mg2c%5Fv2.1/, accessed on 7 April 2025) [49].

### 4.3. Evolution of CsFKBP Protein in Tea

We constructed an evolutionary tree for the *Arabidopsis*, rice, Longjing 43, Shuchazao, and Yunkang10 *FKBP* genes by the neighbor-joining (NJ) method (execution parameters: Poisson correction, pairwise deletion, and bootstrap (1000 repetitions)) using MEGA 7.0 software [50].

### 4.4. Gene Structure and Motif Analysis

The exon/intron structure of each CsFKBP gene was analyzed using GSDS2.0 (http://gsds.gao-lab.org/, accessed on 7 April 2025) to align the coding gene sequence with the corresponding CsFKBP gene sequences retrieved from the Yunkang10 genome [51]. Protein-conserved motifs of CsFKBPs were visualized by setting 10 motifs in the MEME Suite 5.5.7 online tool (https://meme-suite.org/meme/index.html, accessed on 7 April 2025). Gene structures and chromosomal location were analyzed using TBtools-IIv2.154 [52].

### 4.5. Total RNA Extraction and Expression Analysis

We extracted total RNA from frozen tea-tree components, including shoots, roots, young stems, new leaves, old leaves, flowers and buds, using a silica-matrix-column method reagent plant-total RNA extraction kit (TIANGEN, Beijing, China) according to the instructions for use. qRT-PCR reactions were performed using SYBR^®^ Premix Ex Taq TM II (TakaRa, Shanghai, China) in a 20 µL reaction system, using 1 µL cDNA and 1 µL primer, with *CsActin* as an internal reference control. qRT-PCR reactions were programmed under the following conditions: 95 °C predenaturation for 30 s, followed by denaturation at 95 °C for 5 s, annealing at 60 °C for 34 s, and extension at 72 °C for 15 s, maintained for 40 cycles. Primers specific to the CsFKBPs and CsActin used for qRT-PCR analysis are shown in Supplementary Table S1. Three replicates of each PCR run were performed. Relative changes in gene expression were analyzed according to the threshold cycle (Ct) using the 2^−ΔΔCᴛ^ method previously reported [53]. The same method was used for the analysis of FKBP gene expression at different periods under low-temperature treatment conditions.

### 4.6. Gene Cloning and Vector Construction

The open reading frame (ORF) of *CsFKBP53* was amplified using PrimeSTAR^®^ Max DNA Polymerase (Takara, Shanghai, China) with an amplification program of denaturation at 95 °C for 4 min, followed by 33 cycles of 98 °C for 10 s, 58 °C for 15 s, and 72 °C for 10 s. After recovering it using Takara Mini BEST Agarose Gel DNA Extraction Kit Ver.4.0, we then ligated it into the pEASY^®^-Blunt Cloning Vector (TransGen, Beijing, China). The sequencing of CsFKBP53 was performed at Sangon Biotechnology Co., Ltd. (Kunming, China).

### 4.7. Protein Subcellular Localization Studies

We predicted the subcellular location of CsFKBP53 subcellular localization using WOLF PSORT (https://wolfpsort.hgc.jp/, accessed on 7 April 2025) [4]. The full-length sequence of CsFKBP53 with the terminator deleted was ligated into a transient expression vector containing a green fluorescent protein (GFP) reporter gene to generate a 35S::*CsFKBP53*::GFP fusion vector. The recombinant plasmid and the control 35S::GFP were transformed into *Agrobacterium tumefaciens* GV3101. After adding Kanamycin (50 mg/mL) and rifampicin (100 mg/mL), incubate at 28 °C for 24 h and 200 rpm. Centrifuge at 4000 rpm for 10 min when the concentration reaches 1.0. The precipitate was then resuspended with a suspension buffer containing 10 mM MgCl_2_, 10 mM MES, and 100 µM acetosyringone (AS), and OD_600_ was then adjusted to 0.6–0.8. The resuspension was injected into the lower epidermis of well-grown leaves of *N. benthamiana*. The GFP signal was observed using a laser confocal microscope (OLYMPUS, Tokyo, Japan) after 72 h of dark–light incubation. The GFP channel signal was observed at 500–540 nm, while the DAPI and TD signals were at 650–750 nm.

### 4.8. Overexpress and Functional Analysis of CsFKBP53 Under Low-Temperature Treatment

The ORF of CsFKBP53 was ligated onto the overexpression vector P30-3MYC using double digestion, and the plasmid was extracted and transferred into *Agrobacterium tumefaciens* GV3101 using the freeze–thaw method. The transfer into *Arabidopsis* was carried out by flush infestation. In this study, four transgenic positive plants were screened for resistance to Kana on the P30-3MYC vector using Clumbia-0 (Col-0) as the wild type, followed by the extraction of genomic DNA by PCR amplification using 35S and gene-specific primers and validation (Supplementary Table S2). We then analyzed the expression levels of four transgenic strains overexpressing *CsFKBP53* by qRT-PCR. The results showed that *CsFKBP53* was more significantly expressed in the overexpression lines OE-1/6/9. We selected overexpression plants for further analysis and consulted a previous method where transgenic plants were exposed to −6 °C for 2 h and then incubated at 25 °C for 4 d to record survival rates [40].

### 4.9. Physiological Indices of Peroxidase (POD), Superoxide Dismutase (SOD), and Catalase (CAT)

The overexpressed *Arabidopsis* plants and the control group were treated at low temperatures for 24 h. The POD, SOD and CAT activities were measured simultaneously with the normothermic control group, in which the physiological indices of POD, SOD, and CAT were determined according to the specific instructions of the POD, SOD, and CAT activity test kit (Solarbio, Beijing, China).

## 5. Conclusions

The FKBP family has been extensively characterized in diverse plant species, yet a systematic investigation into CsFKBPs in tea plants remains limited. Here, we report the genome-wide identification of 21 *CsFKBP* genes in tea and provide a comprehensive analyses of their evolutionary relationships, gene architectures, conserved protein domains, and transcriptional responses to low-temperature stress. Notably, *CsFKBP53* was highlighted as a promising candidate for enhancing cold tolerance through functional screening. The *CsFKBP53* gene enhanced the cold resistance of *Arabidopsis* plants. However, the research on the genetic transformation system of the tea tree is incomplete, the gene in the tea plant can only be verified by transforming other plants. As the research on the transformation system of tea plants is gradually improved, the target gene can be transferred into tea tree tissues through plant tissue-culture technology, which can be used to further understand the function of the *CsFKBP53* gene, and then breed high-quality tea plant varieties with remarkable cold resistance and excellent tea quality.

## Figures and Tables

**Table 1:** List of *CsFKBP* genes identifies in tea plant.

Gene Name	Gene ID	FKBP-C Domain Number	CDS Length/bp	Amino Acid/aa	Molecular Weight/kD	Theoretical pl	Subcellular Localizations
*CsFKBP11*	CsasTrans141447	1	306	101	11.02	8.87	Nuclear
*CsFKBP12*	CsasTrans179814	1	692	112	11.93	7.75	ChloroPlast
*CsFKBP12-1*	CsasTrans156117	1	332	110	12.26	10.13	Nuclear
*CsFKBP13*	CsasTrans097150	1	1075	244	25.74	8.7	Peroxisome
*CsFKBP15-1*	CsasTrans095919	1	959	153	16.34	7.68	Vacuole
*CsFKBP16-2*	CsasTrans065843	1	1113	238	25.46	9.44	Cytoplasm
*CsFKBP16-3*	CsasTrans159019	1	1132	273	29.67	8.47	ChloroPlast
*CsFKBP16-4*	CsasTrans093053	1	1200	234	24.74	9.68	ChloroPlast
*CsFKBP17-2*	CsasTrans181995	1	1220	259	27.69	6.18	ChloroPlast
*CsFKBP18*	CsasTrans153703	1	1036	237	25.4	9.58	ChloroPlast
*CsFKBP19*	CsasTrans009318	1	1424	295	32.24	6.79	Extracell
*CsFKBP20*	CsasTrans077854	1	2327	188	20.5	7.76	Nuclear
*CsFKBP27*	CsasTrans003218	1	1236	238	27.09	8.64	ChloroPlast
*CsFKBP33*	CsasTrans135390	1	900	300	33.82	4.52	Nuclear
*CsFKBP42*	CsasTrans194578	1	1493	364	41.76	5.66	Nuclear
*CsFKBP47*	CsasTrans171260	3	1920	427	46.72	4.76	Cytoplasm
*CsFKBP53*	CsasTrans084696	1	2329	651	72.43	5.61	Nuclear
*CsFKBP53a*	CsasTrans162628	1	2429	100	10.81	10.44	ChloroPlast
*CsFKBP62*	CsasTrans005298	2	2600	571	63.81	5.17	Peroxisome
*CsFKBP72*	CsasTrans105671	3	2428	619	69.99	5.35	Nuclear
*CsTIG*	CsasTrans154119	0	1151	383	43.06	5.09	Nuclear

## Data Availability

The original contributions presented in this study are included in the article/Supplementary Materials. Further inquiries can be directed to the corresponding author(s).

## Supplementary Materials

The following supporting information can be downloaded at: https://www.mdpi.com/article/10.3390/ijms26083575/s1.
