# Supplementary material for: Genome-Wide Analysis of Tea FK506-Binding Proteins (FKBPs) Reveals That CsFKBP53 Enhances Cold-Stress Tolerance in Transgenic Arabidopsis thaliana
Source: Int J Mol Sci. 2025 Apr 10;26(8):3575. doi: 10.3390/ijms26083575 (PMC12027416; doi:10.3390/ijms26083575)
Supplement: Supplementary file 1 [file ijms-26-03575-s001.zip › ijms-3492844-supplementary.pdf]

**Table S1.** *CsFKBPs* and *CsActin* specific primers for qRT-PCR analysis.

| Primer name | Sequences (5' to 3') |
|---|---|
| *qCsFKBP11-F* | TTCTCGCCCTGGCACATTC |
| *qCsFKBP11-R* | CATTGAGCGGGAACAACCATT |
| *qCsFKBP12-F* | ACCGAGCAACTTCTCCAACCT |
| *qCsFKBP12-R* | GGCAGCAGCCATTCACCTT |
| *qCsFKBP12-1-F* | AACACCAAGATCCCATCCACG |
| *qCsFKBP12-1-R* | CAAGGGTCCCGAAGCAAAA |
| *qCsFKBP13-F* | GGGAAATTGGACAATGGGACG |
| *qCsFKBP13-R* | CCAGCAAGCATGGGAGGAACT |
| *qCsFKBP15-1-F* | GATGGAGGGAATGCAGATGACA |
| *qCsFKBP15-1-R* | ACAATCCTTCGACTCACCCAAA |
| *qCsFKBP16-2-F* | ATCCTTCACTTCCTATTTCCCTTGT |
| *qCsFKBP16-2-R* | AACCTGCTGGCTGCTTTTCA |
| *qCsFKBP16-3-F* | TAACTAAGGTCAACTCATCCCCATT |
| *qCsFKBP16-3-R* | GGCTTCATCTTTACTGTTTCCACTC |
| *qCsFKBP16-4-F* | GGGACTTGGTGTTGGTGGTG |
| *qCsFKBP16-4-R* | TTGTCGCATTTGGAGGGATT |
| *qCsFKBP17-2-F* | GAGAAGCCTGCCAACCCTGT |
| *qCsFKBP17-2-R* | AAGGCGGGTTTTGATTTGTTC |
| *qCsFKBP18-F* | AGACCTTGGGGAGATGGACG |
| *qCsFKBP18-R* | TTGGGGCAGCGAAATGAG |
| *qCsFKBP19-F* | CGCAGCACAAAATCCAAGGC |
| *qCsFKBP19-R* | GATGGGACCAAGACCAACAACA |
| *qCsFKBP20-F* | AAAGGCTATGGCCGACGAA |
| *qCsFKBP20-R* | AGGAGACGGAGAACCTGGAAA |
| *qCsFKBP27-F* | GGGCTTGACAGAGCGGTGAT |
| *qCsFKBP27-R* | GTAAACAGTTGAGTTCGGAGGGA |
| *qCsFKBP33-F* | TGTGGTGGGCAAATCAAACG |
| *qCsFKBP33-R* | CTGAAGCAAAAGTAGGAAAGCGAAT |
| *qCsFKBP42-F* | ATAGCCTAGTTCCCAACCCACA |
| *qCsFKBP42-R* | GGACTGAAAGCACCCAGCACA |
| *qCsFKBP47-F* | ATGGTTCCGTTGGAGATGATGA |
| *qCsFKBP47-R* | AAGCTTGCGCTCGCCGATA |
| *qCsFKBP53-F* | CTTCTCTGTTATTGGTCCTCG |
| *qCsFKBP53-R* | TCTCCTCATCAACCTCTCTGT |
| *qCsFKBP53a-F* | AAGCAAGGGAGCGATGCAG |
| *qCsFKBP53a-R* | TTACCCATTGCCAACTCCTCA |
| *qCsFKBP62-F* | TTCATCGCCATTATCAGGAGTG |
| *qCsFKBP62-R* | GAGAAGGAGATCGGGAAGCAA |
| *qCsFKBP72-F* | TCTGAAGTCGGAACCATTGATAGG |
| *qCsFKBP72-R* | GCCACAACTGCGACATGAAACT |
| *qCsTIG-F* | ATGACCCTTTTGTAACAATCCTCAC |
| *qCsTIG-R* | TCTTCAACCGCTTACGACACC |
| *CsActin-F* | AGGTATGCGAGTTGGTGAC |
| *CsActin-R* | GTTTATGCGAGGGAGTGAA |

**Table S2:** Primers for PCR amplification.

| Primer name | Sequences (5' to 3') |
|---|---|
| 35S | CAAAAGCAAGTTCTTC |
| *CsFKBP53-MYC-F* | agcgaggaggacctgggatcc<br>ATGGCTTTCTGGGGGGTTG |
| *CsFKBP53-MYC-R* | tgcctgcaggtcgactctaga<br>TCAACGGATGCCAACCAATT |
| *CsFKBP53-GFP-F* | atacatatgcccgtcgac<br>ATGGCTTTCTGGGGGGTTG |
| *CsFKBP53-GFP-R* | cccttgctcaccatggatcc<br>TCAACGGATGCCAACCAATT |

**Table S3:** Information on the distribution of CsFKBP genes on chromosomes.

| Gene Name | Gene ID | Chromosome Position | | Chromosome | Chromosome Length |
|---|---|---|---|---|---|
| | | Start | End | | |
| *CsFKBP11* | *CsasTrans141447* | 150397668 | 150402019 | Chr11 | 123678568 |
| *CsFKBP12* | *CsasTrans179814* | 30433377 | 30439782 | Contig235 | / |
| *CsFKBP12-1* | *CsasTrans156117* | 68547213 | 68554705 | Chr01 | 222834174 |
| *CsFKBP13* | *CsasTrans097150* | 68547213 | 68554705 | Chr08 | 163071019 |
| *CsFKBP15-1* | *CsasTrans095919* | 118717972 | 118729386 | Chr11 | 123678568 |
| *CsFKBP16-2* | *CsasTrans065843* | 3660381 | 3663353 | Chr01 | 222834174 |
| *CsFKBP16-3* | *CsasTrans159019* | 119258292 | 119273773 | Chr05 | 194877589 |
| *CsFKBP16-4* | *CsasTrans093053* | 180260602 | 180271998 | Chr10 | 167113155 |
| *CsFKBP17-2* | *CsasTrans181995* | 120469099 | 120512418 | Chr15 | 119080000 |
| *CsFKBP18* | *CsasTrans153703* | 12301918 | 12286232 | Chr04 | 196298966 |
| *CsFKBP19* | *CsasTrans009318* | 1528232 | 1517939 | Chr06 | 181129525 |
| *CsFKBP20* | *CsasTrans077854* | 104889875 | 104881340 | Chr01 | 222834174 |
| *CsFKBP27* | *CsasTrans003218* | 104889875 | 104881340 | Chr11 | 123678568 |
| *CsFKBP33* | *CsasTrans135390* | 159079575 | 159081480 | Chr06 | 181129525 |
| *CsFKBP42* | *CsasTrans194578* | 97768917 | 97777888 | Chr03 | 187390788 |
| *CsFKBP47* | *CsasTrans171260* | 81560624 | 81556237 | Chr04 | 196298966 |
| *CsFKBP53* | *CsasTrans084696* | 81560624 | 81556237 | Chr06 | 181129525 |
| *CsFKBP53a* | *CsasTrans162628* | 81551093 | 81545933 | Chr01 | 222834174 |
| *CsFKBP62* | *CsasTrans005298* | 114762618 | 114761013 | Chr04 | 196298966 |
| *CsFKBP72* | *CsasTrans105671* | 574090 | 568326 | Chr04 | 196298966 |
| *CsTIG* | *CsasTrans154119* | 150397668 | 150402019 | Chr05 | 194877589 |

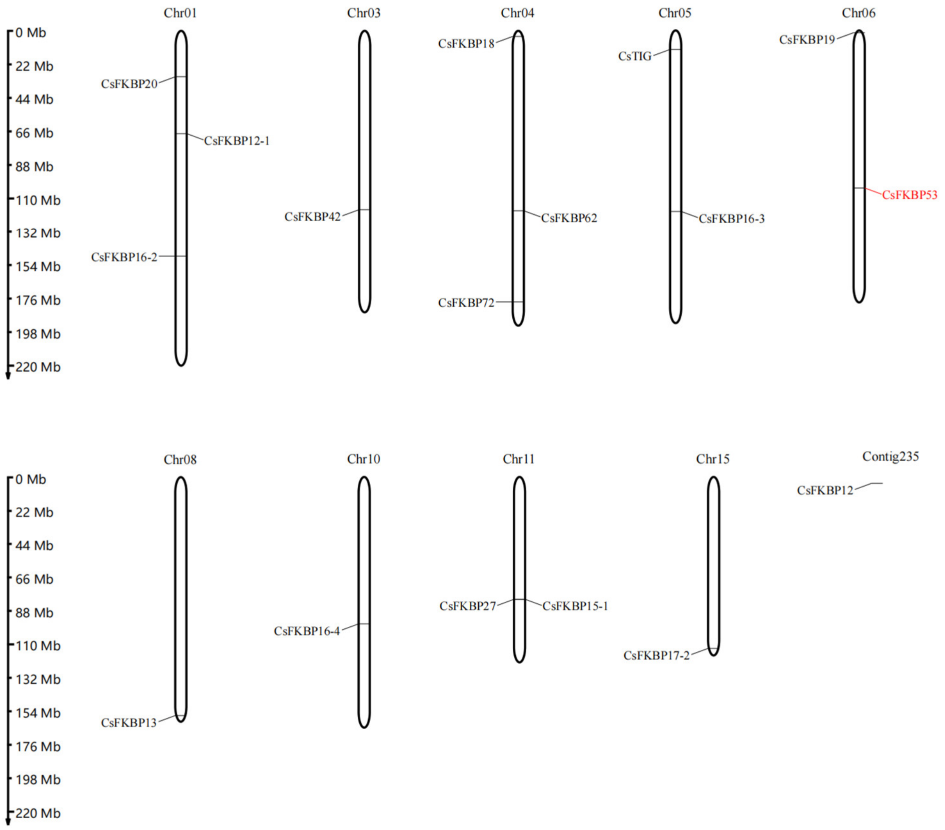


**Figure S1.** Chromosomal location of CsFKBP genes.
